# Medical Clinic, Held by Dr. Francis Delafield, November 14, 1898, at Roosevelt Hospital, New York City

**Published:** 1898-12

**Authors:** 


### MEDICAL CLINIC, HELD BY DR. FRANCIS DELAFIELD, NOVEMBER 14, 1898, AT ROOSEVELT HOSPITAL, NEW YORK CITY.

CASE 1.—*Empyema.*—Gentlemen: The first case I have to show you is a boy, aged seven years, who entered the hospital October 25. Four years ago he had measles. He has also had whooping cough; otherwise he has been well, until he began to be sick September 30. He came into the hospital October 25, when he developed a sense of oppression of the chest, fever and prostration. There was no chill, convulsion, headache, or pain. Three hours later there was a dry, aggravating cough, vomiting and diarrhœa. These symptoms continued four or five days, when the diarrhœa and vomiting stopped. The cough was better but the fever continued. The pain was referred to the epigastrium and was increased by coughing. Two weeks from the onset the patient was better, but only for a short time. Since then he has had great prostration and much cough; his chief complaint is the latter.

This history is entirely indefinite, and no certain conclusions can be drawn from it. When he came into the hospital he was pale, but still fairly nourished; his tongue was coated and moist. The temperature was 102 degrees, pulse 128, respiration 36. The specific gravity of his urine was 1020, and contained no albumin. Over the upper part of the right chest behind there was flatness on percussion; the breathing was rough and high-pitched. Over the lower part there was diminished breathing. Over the right lung in front there was exaggerated voice and breathing. It was uncertain whether the case was one of pleurisy with effusion or empyema; either was possible. Placing a needle in the chest showed that the fluid was pus, so it was evident that the boy had empyema from September 30 to October 25, when he entered the hospital. He was put to bed, given whisky, and his chest was aspirated, 200 cubic centimeters of a thick yellow pus being drawn off. On the 26th of October he appeared better; on the 31st 100 more cubic centimeters of fluid was drawn off. On November 1 came on duty and saw the patient for the first time. I saw no reason for deferring opening the chest, which was done on November 4, when three inches of the seventh rib was removed and drainage introduced. There was a considerable discharge of pus; the day after operation the discharge had fairly changed in character and was serous instead of purulent. After the operation the temperature went down until now it is nearly normal and the boy apparently well. I will now have the dressings changed so that you can see where the drainage is.

This is a good ordinary example of empyema. As a rule they do as well. The point in this case is this, the boy has not only had a certain amount of pus removed from his chest, but the purulent pleurisy has been controlled; it is now no longer a purulent pleurisy; he now has a pleurisy with some effusion of serum. The object of the operation was not alone to empty the chest of pus, but to cure the pleurisy. The little fellow is now in good shape.

CASE 2.—*Influenza.*—This next patient is a man 22 years of age. He is an Italian, and was admitted to the hospital on the 8th of November. Two weeks previous to this date he was suddenly taken sick with a chill, sharp pain in the right side which was increased upon coughing. The patient took to bed at once, remaining in bed until he came to the hospital. He continued to have pain in the right side. There was no expectoration of blood. He complained principally of cough and the pain in the side. When he entered his temperature was 98.4, pulse 108, breathing 26. His tongue was thickly coated. His breathing was rapid and shallow. His abdomen was distended and tender. His face was apathetic and the man looked sick, although he only had a temperature of 98.4. Over the entire right lung behind there was a subcrepitant friction sound; this was the only physical sound obtained, although he was expectorating a muco-purulent fluid. There were no coarse rales. The next morning after admittance his temperature was 102 degrees, the next, 99 degrees, the next day 102 degrees in the afternoon, next morning 102 degrees; on the 11th of November it was 101 degrees, and to-day it is down between 99 and 100 degrees. So when he first came in, the temperature ranged from 99 to 102 degrees; it now ranges from 99 to 100 degrees. The cough continued and there was an abundant muco-purulent expectoration. The evidence is that he has bronchitis with a muco-purulent expectoration. He was sick for two weeks before his entrance to hospital, which makes three weeks that he has been sick altogether. The tongue has not entirely cleared off, and the expression of his voice has changed.

This is a straight history of one of the severer forms of epidemic influenza, and is the first one I have seen this fall. This case is a good one to give you an idea of how much of a disease an attack of epidemic influenza is. This man has fever which belongs to it; how high it was at first I do not know; at the end of three weeks the fever had only just gotten down between 99 and 100 degrees, which is one of the features which belong to this disease. He neither gave history of pain in back or limbs which are so often the marked features. Inflammation of the nose, throat or larynx have not been present. Inflammations of the bronchi have been present. There has been no asthma or coarse rales or dyspnoea. He has had also a dry pleurisy, which belongs to some cases of influenza. The effect of the poison upon the appearance of the patient is fairly well marked; when he came in at the beginning of the third week he looked like a man with typhoid fever, and these patients are often supposed to have typhoid fever. The patient is now fairly convalescent. The thing now to do is to get rid of his bronchitis. He has been given ipecac for his bronchitis, but we will now place him upon constant inhalations of creosote. He no longer needs to be in bed.

CASE 3.—*Aneurism of the Arch of the Aorta.*—This woman is 48 years of age, and was admitted to the hospital on October 31. Her general health was good until history of this illness appeared. Five years ago after exposure to wet and cold, she had chilly feeling every night and perspired profusely. Later she felt a sharp stabbing pain to the left of the sternum, which was followed by cough. Ever since that time, whenever she caught cold, there has been a repetition of this sort of an attack with pain and chillness. There was a dry cough and a slight mucous expectoration; these symptoms occurred every two or three weeks. Otherwise she has not been sick.

About one year ago her cold was a little worse and she stayed in bed; there was considerable expectoration. Eight months ago there was a shortness of breathing upon exertion and some change in the voice. This dyspnea on exertion and loss of voice has been progressive. During the past month her cough has considerably increased, with increase of expectoration. There has been no pain on coughing nor any hemoptysis. She has a certain amount of pain at night, but her chief complaint is the cough.

Now, for purposes of diagnosis, I shall omit all history up to eight months ago, which was the date of her real illness. Now observe that during the eight months the main symptoms have been cough, change in the patient's voice, shortness of breath on exertion, and increase in the amount of expectoration. The change in the voice is not so much in its quality as in its strength. She has not the appearance of a sick woman. Her tongue is clean, her pulse is full and regular, yet she came in with temperature of 98 8 degrees, breathing 24, and her urine showed a specific gravity of 1020, with no albumin. She is now breathing quietly, she has no temperature, and there is nothing about her to indicate that she is a particularly sick woman.

The physical signs show good resonance over the front of chest; over the left lung in front there is a marked dullness on percussion, and also tenderness. In the axillary region there is flatness and loud gurgling rales; it is normal on the right side. Please note that there is on the left side, from the clavicle down, marked dullness on percussion, changing to flatness in the axilla and loud gurgling rales. Over the upper part of the left lung behind there was complete flatness on percussion. There is loud tubular breathing. There is no change in the heart's position, nor any murmur. These physical signs practically existed up to the present time. Note that the flatness is complete and extends well down the whole distance of the lung behind. Now to make a more explicit diagnosis, there has been introduced a needle in the left side of the chest and no fluid has been found.

Now, gentleman, here is a woman with no fever, pretty well nourished, apparently feeling well, with a history of eight months' dyspnoea on exertion, cough, increase in expectoration and change in character of the voice. The physical signs, to repeat, show the right lung normal, but the whole of the left lung behind, flatness on percussion, with loud tubular breathing. In the axillary region there is marked flatness and loud gurgling rales. Now what can account for all this? What is the condition of the left side of the chest?

One should think of aneurism. But this woman gives none of the ordinary signs of aneurism; there is nothing wrong with the heart at all. If the large bronchi of the lung are obstructed you can get, over the front of the lung, flatness on percussion; this is true. This may occur from pressure by an aneurism or any new growth, or in the curious cases of rapid bleeding into the bronchi, so that these bronchi are completely blocked up by the large clots formed. In this way we can account for the flatness on percussion. In the same way the pressure on the bronchi capable of giving tubular breathing and rales, without any change in the lung itself outside of the pressure. This woman has an aneurism, given off at the origin of the aorta, which is pressing on the large bronchi, so producing obstruction, which readily accounts for the dyspnoea and physical signs. Since she has been in the hospital she has been kept quiet and given large doses of iodide of potassium; under his treatment the dyspnoea has become less and the woman feels better in every way. To make the diagnosis more complete there is the additional sign of pulsation which can be felt at times only. There is no question about our diagnosis; it is an aneurism of the arch of the aorta at the base of the lung.

I think if any one would simply listen to her chest without looking at her, would say that the upper lobe of the left lung was riddled with cavities, that she had had an old tubercular inflammation, with cavity following, etc. Yet the moment one looks at this woman you can see that this would be impossible, to have her lung in that condition and appear as she now appears.

CASE 4.—*Acute Rheumatism.*—This man is 47 years of age. He entered the hospital on the 28th of October. Seven years ago he had a moderate attack of rheumatism in the right knee, elbow and hand. Since he has been well, until the 24th of October, when he began to have pain in the right hand with a little swelling. On the 26th of October the elbow and shoulder were swollen and tense. On the 28th he had a severe sharp pain with swelling in the knee, and all the joints became swollen and painful. This was the history of an invasion of an acute attack of articular rheumatism, beginning on the 24th of October with the first symptoms of synovitis. This occurred four days before he was admitted to the hospital. When he came in his nutrition was good. He was a large, florid man. His tongue was moist and coated. His pulse was 120, full and regular. The right hand, knee, elbow and shoulder gave the ordinary appearances of rheumatic synovitis, the parts being swollen, painful and red. The temperature was 99 degrees, pulse 120 and respirations 26. There were no constitutional disturbances. The specific gravity of the urine was 1022, and contained a few granular casts. The heart was all right. On the 28th of November he was placed on salicylate of soda, grains 20, in solution, every three hours. That is the ordinary dose we give for cases of acute rheumatism. He was placed in bed and given a milk diet. The local dressing consisted of oil of wintergreen placed about the inflamed joints. On the 29th the left elbow became red and tender; the other joints were about the same. On the 31st the left foot, knee and hand were all badly inflamed. On November 1 the synovitis was not any better. The quantity of salicylates was increased, which caused some ringing in the ears, but did not make the rheumatism any better. On November 1 he began to show some constitutional disturbances and was a little delirious. The temperature chart shows fever had gone up. The highest point reached was 104 degrees; the temperature kept on going up while I was using the salicylates; the 104 degree point was reached on November 1. So here was a man who was treated for four days on salicylate of soda, and yet not getting any better, but getting worse. When this occurs it means that the salicylate is not the drug for that particular case of rheumatism, and that under its use the patient will not get well. One must then stop it and try some other plan of treatment. In this case we had the poison of the salicylates and the poison of the rheumatism. It is a rule that if we get a poisonous effect from the drug administered it does not have any specific effect on the poison of the disease. So having tried it, stop it and place the patient upon the old alkaline treatment, as I did in this case, giving him one dram of bicarbonate of potassium in lemon-juice four times a day. The next day sweating occurred and the patient felt better. I then increased the bicarbonate of potassium to two drams in lemon-juice four times a day. By the 6th of November the urine as rendered alkaline and has kept so ever since. This was begun on the 1st of November; on the 2d he was better and the temperature was found to be going down; on the 3d, 4th, 5th, 6th and 7th it kept falling, when it got down to 99 degrees, and has been to 98 degrees. The synovitis has disappeared and the man is now fairly convalescent from his rheumatism. There has been no heart complications. This is a straightforward example of the fact that there are certain cases where it is best to administer the old alkaline treatment; this man belongs to just that class of patients—strong, red, florid men who do better under this plan of treatment. This man has really done a little better than they usually do; he has had a temperature of 104 degrees, all his joints inflamed and was a very sick man, and it only took four days of the alkaline treatment of two drams of bicarbonate of potassium, effervescing in lemon-juice, to practically cure him.—*Medical Times.*

